# The Challenges and Opportunities of Next-Generation Rotavirus Vaccines: Summary of an Expert Meeting with Vaccine Developers

**DOI:** 10.3390/v14112565

**Published:** 2022-11-19

**Authors:** Jessie Chen, Stephanie Grow, Miren Iturriza-Gómara, William P. Hausdorff, Alan Fix, Carl D. Kirkwood

**Affiliations:** 1 Enteric & Diarrheal Diseases, Bill & Melinda Gates Foundation, Seattle, WA 98109, USA; 2 IVES, University of Liverpool, Liverpool L69 7BE, UK; 3 PATH, 1202 Geneva, Switzerland; 4 Faculty of Medicine, Université Libre de Bruxelles, 1050 Brussels, Belgium; 5 PATH, Washington, DC 20001, USA

**Keywords:** rotavirus, vaccines, next generation, combination vaccines

## Abstract

The 2nd Next Generation Rotavirus Vaccine Developers Meeting, sponsored by PATH and the Bill and Melinda Gates Foundation, was held in London, UK (7–8 June 2022), and attended by vaccine developers and researchers to discuss advancements in the development of next-generation rotavirus vaccines and to consider issues surrounding vaccine acceptability, introduction, and uptake. Presentations included updates on rotavirus disease burden, the impact of currently licensed oral vaccines, various platforms and approaches for next generation rotavirus vaccines, strategies for combination pediatric vaccines, and the value proposition for novel parenteral rotavirus vaccines. This report summarizes the information shared at the convening and poses various topics worthy of further exploration.

## 1. Introduction

Diarrheal disease continues to be a major cause of global morbidity and mortality among children under 5 years of age. In 2019 alone, over 500,000 deaths and over 45 million disability-adjusted life years (DALYs) were estimated to be attributable to diarrheal diseases (9.92% and 9.66% of all causes, respectively) [1]. Rotavirus remains the major cause of severe viral diarrhea, despite the development of four live attenuated oral rotavirus vaccines (LORVs) with WHO prequalification (Rotarix, Rotavac, Rotasiil, and RotaTeq), and a WHO recommendation for rotavirus vaccine inclusion in all national immunization programs, resulting in their use in over 120 countries [2]. However, despite this progress in vaccine development and introduction, a substantial number of countries have yet to introduce LORVs, and LORVs have been demonstrated to be less effective in preventing severe gastrointestinal disease in low-income countries in comparison to high income countries [3,4,5,6,7].

The goal of next-generation rotavirus vaccines is to improve the effectiveness and impact of rotavirus vaccination programs in low-income countries (LIC) and lower middle-income countries (LMIC), by further reducing morbidity and mortality associated with rotavirus infection and associated moderate to severe diarrhea (MSD). At the time of this meeting, only one next-generation rotavirus vaccine candidate has been under evaluation in late-stage clinical trials. PATH has been advancing a parenteral vaccine candidate, the P2-VP8 (P[8], P[6], P[4]), a trivalent non-replicating rotavirus vaccine (commonly referred to as NRRV) [8] over the last decade, through to current phase 3 clinical testing [9]. However, given the importance of addressing diarrheal disease burden, it is critical to identify and accelerate the development of other candidates to ensure a robust pipeline of next generation vaccines, with the aim to improve efficacy, impact, and to ensure sufficient vaccine supply and choice (Table 1). This meeting was convened to provide an update on the status and challenges surrounding rotavirus burden, as well as to identify and highlight the gaps remaining in our understanding of rotavirus immunology, advances in development of next generation vaccine candidates and the value proposition and appeal of these candidates, and advantages as well as challenges in development of a combination rotavirus vaccine approach.

## 2. Rotavirus Burden and Challenges

Carl Kirkwood (BMGF) opened the meeting with an overview of the impact and status of current oral rotavirus vaccines. Rotavirus vaccines have been introduced into national immunization programs of 120+ countries across the globe [9], leading to significant reductions in rotavirus-related morbidity and mortality across all high-, middle-, and low-income settings. Reductions in rotavirus gastroenteritis (GE) associated hospitalizations and/or emergency department visits ranged from 28–46%, depending on age group and overall child mortality rates of the setting [10]. For example, in India where Rotavac was introduced in April 2016 and Rotasiil in April 2018, and rolled out country wide, the proportion of diarrheal-related hospitalizations attributable to rotavirus declined from 46.2% in 2014 to 13.3% in 2019 [11].

Several new live oral vaccine candidates are under development. The neonatally orally administered RV3-BB (PT Bio Farma) is currently in a phase 3 efficacy study in Indonesia. If successful, Indonesia plans to seek regulatory approval and introduce the vaccine into the national immunization program in 2023/2024. The UK-BRV oral vaccine, licensed to manufacturers including Wuhan (China), Butantan (Brazil), and Serum Institute of India (in the form of Rotasiil), has completed phase 3 clinical trials in China. Rotavin-M1^®^ and Rotavin (liquid formulation) (POLYVAC) have been licensed in Viet Nam, with an effectiveness study of the impact of the vaccine in 2 provinces nearing completion. The LLR oral vaccine (Lanzhou Biologicals/Xinkexian Biological Technology) is licensed in China, with the trivalent LLR3 also having completed phase 3 clinical trials in China [12]. Despite the impact and ongoing development programs, 62 countries remain without plans for LORV introduction into national childhood immunization programs.

Veronica Denti (Gavi) presented on the challenges facing rotavirus vaccine supply, and the vaccine choice landscape. Gavi has supported the introduction of LORVs to 53 countries since 2008, with Nigeria, Bangladesh, Central African Republic, and Viet Nam anticipating introductions in 2022 or 2023 [9]. There are oral rotavirus vaccines available for Gavi-eligible countries from three manufacturers: GSK (Rotarix), Bharat Biotech of India (Rotavac), and Serum Institute of India (Rotasiil). A total of 12 presentations were supported by Gavi, of which eight were available at the time of this meeting. Supply constraints have been a consistent challenge in recent years due to manufacturers either reducing vaccine allocation to Gavi or having production issues, resulting in several countries being forced to switch vaccine products.

Additionally, many countries have limited capability or information to follow a systematic, evidence-based process for product selection. Support tools for evidence-based country decision-making are central to assisting countries in optimizing their vaccine choice. Measures to support country decision making process could include: (1) capacity building of national immunization technical advisory groups (NITAGs) and expanded programs on immunization (EPIs) through standardizing webinars and technical resources (such as the PATH Rotavirus Vaccine Cost Calculator) to help countries be aware of and assess vaccine options, (2) repeated dissemination of evidence through a central information repository, and (3) improved engagement of Gavi and Alliance partners with NITAGs in priority countries. Investments in country awareness before new vaccine prequalification could allow countries to be better equipped to make informed and rapid decisions once a new vaccine becomes licensed and available for use.

However, vaccine availability is only part of the challenge; there are clear differences in LORV effectiveness in low versus high mortality settings, with better performance in low mortality countries; furthermore, waning protection in the second year of life is more pronounced in high mortality settings [7,13]. Miren Iturriza-Gómara (PATH) presented on the challenges and strategic approaches for the control of rotavirus disease based on scientific and scheduling factors. A number of approaches to improve LORV immunogenicity have been investigated, including increased vaccine dose, separating LORV administration from oral polio vaccine, co-administering zinc and probiotics, withholding breastfeeding prior to administration, and prenatal nutrient supplementation, but unfortunately none have been shown to have a significant benefit [14,15,16,17,18,19]. One factor shown thus far to be predictive of increased protection from disease is asymptomatic neonatal virus exposure, and clinical studies have indicated that RV3-BB may be more effective in a neonatal schedule compared to a schedule beginning at 6 weeks of age or later [20,21,22]. However, given vaccine effectiveness heterogeneity across LICs and LMICs, it is unclear if the existing WHO PQ vaccines may be safely and effectively used in a neonatal schedule.

The approach taken by most next-generation vaccine developers has been to develop injectable vaccines, which bypass the gut environment that is potentially responsible for poor vaccine effectiveness in LICs and LMICs. In addition, these are likely to have lower cost of goods, a superior safety profile due to the unlikely association of intussusception and have the potential to be combined with other injectable vaccines, reducing costs further and facilitating increased coverage and access.

## 3. Progress with Non-Replicating Rotavirus Vaccine Candidates

Alan Fix (PATH) provided an overview of the trivalent rotavirus P2-VP8 subunit vaccine (NRRV) program. The NRRV was initially developed at the United States National Institutes of Health (US NIH) by Dr. Yasutaka Hoshino and combines recombinant VP8 subunit proteins from strains with P[4], P[6], and P[8] VP4 genotypes, each fused to the P2 T-cell epitope of tetanus toxin. The vaccine is adsorbed to aluminum hydroxide and administered parenterally as a 3-dose regimen. A phase 1/2 immunogenicity study of the trivalent vaccine was completed in December 2017 [23], supporting advancing to phase 3 testing. The ongoing phase 3 efficacy study is a double-blind, randomized active comparator (ROTARIX), multi-country study to assess safety and efficacy of the trivalent vaccine to prevent severe rotavirus diarrhea (NCT04010448). The phase 3 study commenced recruitment in 2019 in Zambia, although enrollment was halted in March 2020 due to the COVID-19 pandemic, and all participants were unblinded and vaccinated with ROTARIX. The study was resumed in March 2021 in Ghana, Malawi, and Zambia and has two stages, the first of which enrolled ~3730 infants, with an interim assessment of futility in regard to demonstrating superior efficacy to Rotarix before progressing to the second stage. Stage 2 was planned to complete full study recruitment (8200 participants). Unfortunately, after this meeting the NRRV vaccine candidate met futility criteria at interim analysis. The study investigators are currently undertaking appropriate subject follow-up, cross over vaccination and commencing wind-down activities.

A phase 2 study to assess the impact of concomitant NRRV and LORV administration and use of NRRV with LORVs in a prime/boost model is ongoing (NCT04344054). Two cohorts of live oral vaccine have been enrolled, one to evaluate NRRV with RV3-BB and a second to evaluate NRRV with ROTARIX. All arms across the two cohorts are challenged with ROTARIX 4 weeks after vaccine series completion to assess reduction in viral shedding. Enrollment for this study was completed on 12 April 2022, with the clinical phase anticipated to be completed in Q4 2022, and top-line immunogenicity results anticipated in Q3 2023.

Sandro Roier (CureVac SE, Germany) presented on the mRNA VP8 vaccine program. A P2-VP8 mRNA vaccine with optimized non-coding regions generated higher IgG1, IgG2a, and P[8] neutralizing antibody titers in mice than the corresponding protein-based subunit vaccine. Importantly, an mRNA-encoded fusion of lumazine synthase from *Aquifex aeolicus* to a slightly longer version of VP8 (LS-P2-VP8) resulted in the formation of self-assembling nanoparticles in vitro and enhanced levels of anti-P[8] IgG and virus neutralizing titers in guinea pigs. In this experiment, monovalent LS-P2-VP8 of genotype P[8] elicited functional antibodies against both homotypic P[8] and heterotypic rotavirus strains P[4] and P[6]. Moreover, a trivalent LS-P2-VP8 mRNA vaccine candidate encoding genotypes P[8], P[6] and P[4] of VP8 induced higher neutralizing antibody titers against P[8], P[6] and P[4] rotaviruses than monovalent LS-P2-VP8 mRNA vaccines. In a gnotobiotic pig model that mimics human rotavirus infection and diarrhea, the trivalent LS-P2-VP8 mRNA vaccine proved to be highly immunogenic. This vaccine improved immunogenicity and reduced viral shedding after challenge to the greatest extent compared to trivalent mRNA vaccine encoding for P2-VP8 alone or the corresponding protein vaccine.

Baoming Jiang (US CDC) presented data on an inactivated rotavirus vaccine (IRV) candidate, derived from a human G1P[8] strain termed CDC-9. Parenterally administered CDC-9 has shown protection in gnotobiotic piglets following oral rotavirus challenge, and basic research undertaken by Stephen Harrison (Harvard University, USA) and Harry Greenberg (Stanford University, USA) has provided insight into the potential mechanism of protection of this strain. A human monoclonal antibody that binds to a region of VP4 where both VP8 and VP5 lie was identified. Interestingly, this antibody effectively neutralizes human rotavirus but does not appear to bind to the blood group antigen binding region of VP4; it may instead block penetration of the virus after it is bound.

Jiang’s group has been evaluating the combination of CDC-9 with inactivated polio vaccine (IPV). Co-intramuscular (IM) administration of IPV and IRV does not result in a reduction in CDC-9 immunogenicity in rodent models, overcoming one of the potential issues facing current LORVs and oral polio vaccines. Recent data also indicate that using a microneedle patch formulation of CDC-9 with IPV does not show immune interference in rodents, and this formulation may in fact be dose sparing for IRV and IPV [24]. Toxicity studies of IM IRV in rats and guinea pigs show no observed local or systemic adverse effects as well as induction of high titers of neutralizing antibodies. A pre-IND meeting request and briefing package (including both the IM and a microneedle patch formulation of CDC-9) have been submitted to the FDA. The design and protocol have been prepared for a phase 1 clinical trial of the IM and microneedle patch formulations in the USA, with plans to partner with manufacturers for further phase 2 and 3 trials in countries with high diarrheal disease burden.

Tingdong Li (Xiamen University, China) presented data on a truncated VP4 (VP4*) vaccine candidate. VP4 (the precursor to VP5 and VP8) mediates the attachment and internalization of rotavirus, and therefore its neutralization is hypothesized to provide protection. When compared to VP8, a truncated version of VP4 (aa26-476) containing the VP8 and the VP5 antigen domain has been shown to induce higher immune responses and protective efficacy in mice [25]. An adult mouse challenge model demonstrated VP4* confers 100% protection against rotavirus shedding. Interestingly, VP4* immunogenicity is maintained in the presence of murine maternal antibodies, unlike following immunization with LORV in humans [26,27].

VP4* stimulates higher levels of protective immune responses in animal models, compared to P2-VP8 (unpublished). Additionally, VP4* proteins of P[8], P[4], and P[6] human rotaviruses stimulate high levels of homotypic and heterotypic neutralizing antibodies in guinea pigs and rabbits [28]. Currently, a trivalent vaccine based on a truncated VP4* is under development in partnership with Innovax. Process development is expected to be completed by the end of 2022, with a pre-IND application target of Q3 2023, and Phase 1 trial initiation in Q3 2024.

Ming Tan (Cincinnati Children’s Hospital, USA) discussed progress on a virus-like particle-based multivalent rotavirus vaccine. To enhance the immune response to VP8, the group used norovirus (NoV) capsid-based S_60_ and P_24_ nanoparticles to display the VP8 antigens [29]. The S_60_ and the P_24_ nanoparticles are self-assembled by 60 shell (S) and 24 protruding (P) domains of norovirus capsid, respectively. Immunization using a S_60_-VP8 pseudovirus nanoparticle (PVNP) induces higher VP8-specific IgG titers compared to free VP8 antigen, and reduces rotavirus shedding after murine rotavirus challenge [30]. Moreover, a trivalent rotavirus candidate consisting of PVNPs displaying P[4], P[6], and P[8] antigens elicits high IgG and neutralizing antibody titers to all three VP8s. The group has also developed a method for large-scale production of tag-free PVNPs, first by precipitating target proteins from bacterial lysate using ammonium sulfate, then further purifying using anion exchange chromatography. This has provided a low cost production approach for PVNPs displaying rotavirus antigens of P[4], P[6], P[8], and P[11] genotypes [31]. The group is now focused on developing a new S_60_-based PVNP to display a truncated VP4 (including VP8 and VP5) for further improved immune response and heterotypic protective efficacy.

The ability of peptide nanoparticles to display multiple antigens may be capitalized upon to also protect against other enteric viruses. Using the P-domain of norovirus as the display base for the rotavirus VP8 protein (P_24_-VP8), a high IgG response against both rotavirus VP8 and the NoV P domain was elicited in mice [32]. Further, post immunization sera demonstrated neutralizing capability against rotavirus replication and blocked NoV virus-like particle binding to receptors. Administration of P_24_-VP8 also protected mice and gnotobiotic pigs against a rotavirus challenge [33]. The P_24_-VP8 nanoparticle vaccine candidate may be upscaled for production by a similar method to S_60_-VP8 for the rotavirus types P[4], P[6], and P[8], see [32].

Continuing the theme on multi-viral pathogen protection, Mario Barro (GIVAX Inc., USA) discussed recent work on the use of reprogramming live attenuated rotavirus to express exogenous antigens, including norovirus using a reverse genetics approach. Based on a technology developed at John Pattons’s laboratory in Indiana University, rotavirus dsRNA gene segment 7 can be modified to express proteins without interfering with the viral cycle, and early in vitro studies have shown that an adapted SA11 simian rotavirus can express spike proteins from SARS-CoV2 [34]. Early work has been undertaken to express the NoV VP1 and P protein in a similar manner. Currently, the group is working towards preclinical proof of concept, with the goal of developing an improved oral rotavirus vaccine that also provides protection from norovirus.

## 4. Scientific Gaps in Knowledge and Tools for Future Vaccine Development

Although there are a number of new vaccine candidates under development, significant gaps in our knowledge of rotavirus immunology and pathogenesis remain. As Miren Iturriza-Gómara (PATH) presented, protection against rotavirus disease in humans is not always correlated with the presence of type-specific or cross-reactive antibodies. During natural infection, most neutralizing antibody response is directed against VP4, although more recent work has shown that there are heterotypic neutralizing responses to VP7, VP8, and the VP5 spike stalk region [35,36]. Vaccines, however, have shown different patterns. The LORV neutralizing antibody responses are directed primarily against VP7 [37]. Recent evidence shows that while the absence of VP8-binding may be a superior predictor of disease in unvaccinated children, anti-VP8 antibodies are poorly induced in vaccinated children, and no threshold has been identified for clinical protection [38].

It is highly likely that parenteral rotavirus vaccines will have different pathways to protection and thus generate distinct correlates of protection (CoP) compared to their live oral vaccine counterparts. Because LORVs replicate in the gut, immunoglobulin A (IgA, mostly directed against the inner virus capsid protein VP6) is used as an imperfect correlate of protection. However, it does not capture a true clinical endpoint and does not accurately predict individual levels of protective immunity [39,40,41,42,43,44]. For parenteral vaccines, IgA is not a suitable marker of immunogenicity. Rather, IgG is more likely to be a marker or CoP; transplacental-derived IgG protects infants from rotavirus disease and interfere with LORV take, and hyperimmune serum has been shown to protect non-human primates upon challenge [27,45]. The IgG can also cross epithelial barriers by receptor-mediated transcytosis and has been shown to protect against other viruses in the lung and intestinal lumen [46,47]. Moving forward, studies need to be conducted to understand the role of binding versus neutralizing antibodies in driving protection, identify the key immunogen(s) responsible for broad and durable protection, determine a mechanistic CoP, and standardize assays according to vaccine approach.

The infant controlled human infection model (CHIM) is one tool being developed to assist rotavirus vaccine development, as presented by Nick Grassly (Imperial College of London, UK) on behalf of Roma Chilengi (CIDRZ, Zambia). A CHIM could allow for early optimization and candidate selection studies and may provide important information on immunogenicity and potential CoPs. Currently, a study of 720 infants is being conducted at CIDRZ and is evaluating Rotarix as a challenge rotavirus strain after administration of either the standard Rotarix schedule, a parenteral schedule (P2-VP8), or two alternative combination parenteral/oral schedules. Prevention of subsequent Rotarix viral shedding in stool is being used as a surrogate measure of protection. There are many advantages to this CHIM approach: demonstrated safety, wide availability as a product, an uncomplicated regulatory and ethical framework, applicability to the population and age-group of interest and demonstrated feasibility in LMICs. Limitations, however, include that CHIMs model infection rather than disease, and that a high-dose challenge is not necessarily akin to natural exposure to wild-type strains. Results of this study are expected by August 2023.

Another model for vaccine evaluation may be human intestinal enteroid cultures (HIEs), which are generated from biopsy tissue from infants or adults and contain all epithelial cell types. Sasirekha Ramani (Baylor College of Medicine, USA) spoke on the ability of HIEs to represent the physiological consequences of rotavirus infection, and their potential to serve as a pre-clinical platform to study host factors that affect response to vaccination. One example is modeling the interference of oral poliovirus vaccine on oral rotavirus vaccine response. Similar to what has been shown in vaccine studies, HIEs show that co-infection with Rotarix or Sabin poliovirus type I (mOPV1) results in reduced rotavirus vaccine titers compared to Rotarix infection alone. However, poliovirus titers during co-administration were not impacted. Ongoing studies are characterizing the mechanisms of interference and evaluating strategies to overcome the inhibitory effect of mOPV1 on Rotarix replication.

For inactivated or subunit rotavirus vaccines, HIEs may serve as a tool to screen for potential CoPs. Samples from a phase 1 immuno study of the monovalent P2-VP8 P[8] showed that vaccine recipients have higher histo-blood group antigen (HBGA) blocking antibodies compared to placebo recipients. Titers were also higher in vaccine non-shedders compared to shedders, as well as in infants with a 4-fold rise in neutralizing antibodies to the Wa (homologous) rotavirus strain—indicating these antibodies may be of interest. Past work has shown that for another enteric virus (norovirus), neutralizing antibody levels as defined by HIE cultures correlate with those from HBGA-blocking assays [48]. This suggests that for rotavirus, HIEs may be used as a high-throughput assay to find a functional protective antibody.

Harry Greenberg (Stanford University, USA) presented on several rotavirus (RV) biology topics, including the ability to label RV with luciferase to follow its movement and location during enteric infection in a small animal model. As certain RV strains can spread systemically and cause disease ex gut, this tool may advance our understanding of RV-associated disease. Greenberg then presented evidence on how RV could also be used to assist in a multi-pathogen immunization strategy; for example, the NoV VP1 or P particle can be inserted into the NSP3 encoding gene of RV. Oral with or without intra-peritoneal booster immunization of suckling mice using a rhesus RV-vectored NoV vaccine induced systemic IgG and IgA against RV, as well as varying levels of IgG and IgA against NoV VP1. Follow up studies demonstrated that local enteric IgA responses to RV and NoV were also detectable in the feces following immunization. Of note, serum neutralizing antibody activity to NoV was also detected using either organoid culture or HBGA blocking type assays.

## 5. Value Proposition and Appeal of a Next Generation Parenteral Vaccine

The public health value proposition of any newly developed vaccine must be clearly understood and articulated to the appropriate stakeholders to help guide vaccine design, clinical testing, and ultimately improve the probability of widespread adoption and introduction. Bill Hausdorff (PATH) presented on the public health value of injectable next-generation rotavirus vaccines (iNGRV). Two questions were initially addressed to help focus subsequent analyses of the potential health impact and cost effectiveness of iNGRV, and to gauge preferences among national stakeholders in a range of target countries. These included: (1) To maximize lives saved, should iNGRV studies be focused on improving protection in infants, or be used as a booster dose to extend protection in the second year of life? (2) If a combination with other vaccines is desired to minimize the number of parenteral injections, should an iNGRV be combined with DTP-pentavalent (DTwcP-Hib-HepB), DTP-hexavalent (including inactivated polio vaccine (IPV)), or with IPV alone?

Regarding the first question, an additional booster dose for LORVs would be unlikely to have a major impact on childhood mortality. There is a decrease in LORV efficacy in high-burden countries during the second year of life, with studies showing a range of 23.7–42.2% difference in effectiveness between the first and second years. However, a large amount (~40%) of this waning may be due to age-related accumulation of naturally infected children [49]. Furthermore, the highest incidence of rotavirus-induced mortality is in young infants. Consequently, increasing vaccine efficacy by 50% using a booster at 12 months would be expected to prevent ~4800–7300 additional deaths annually in Africa and SE Asia. While not insignificant, an “effective booster” could only prevent 4–11% of the rotavirus deaths estimated to still be occurring with current infant vaccination programs. In other words, impact would come too late for maximal effect—and therefore future efforts should focus either on increasing effectiveness or uptake of a primary vaccination series.

To minimize added injections while maximizing uptake of an iNGRV, modeling by Linksbridge to anticipate demand for DTP-pentavalent, DTP-hexavalent, and standalone IPV over the next several years suggests that a vaccine that includes iNGRV and either DTP-pentavalent or DTP-hexavalent would be more likely to be widely adopted than iNGRV combined with standalone IPV. However, combination vaccines may command a price premium over co-administration of the standalone vaccines. Therefore, it is important to assess the economic impact of different iNGRV scenarios.

Fred Debellut (PATH) assessed the impact and cost-effectiveness of multiple iNGRV options at varying levels of efficacy and scheduling scenarios (standalone, DTP combination, co-administration with LORVs), assuming 137 LMICs pay full vaccine price (i.e., including the portion currently paid by Gavi) [50]. Over 10 years, a highly efficacious iNGRV would be expected to avert an extra ~200,000 deaths and ~70 million cases in comparison to current LORVs. A standalone iNGRV is expected to be more cost effective than LORVs, even with equivalent efficacy. An iNGRV-DTP combination would be cost-saving in all LMICs even with equivalent efficacy to current LORVs. Given the expected lower price of an iNGRV in comparison to LORVs, use of iNGRV over 10 years could generate economic cost savings between USD $1–15 billion, while an iNGRV-DTP could generate $8–23 billion in savings.

Understanding country preferences beyond that of cost-efficacy for an iNGRV is critical for the development process and to understand potential future uptake. John Bawa (PATH) spoke on a multi-country study conducted to understand country stakeholder preferences [51]. Seventy-one national stakeholders from six countries (Ghana, Kenya, Malawi, Peru, Senegal, and Sri Lanka) were interviewed to seek their preferences given different vaccine formulation, effectiveness, and administration scenarios. Approximately half of the stakeholders preferred a highly efficacious standalone iNGRV to LORVs, but that proportion is cut if efficacy is equivalent. However, a combination iNGRV-DTP vaccine garnered significant support over LORVs, even in the event of equivalent efficacy, because no additional injections or administrations would be required, and cold chain costs would also be reduced. When queried about preferences for neonatal dosing of an oral next-generation rotavirus vaccine, stakeholders expressed a strong preference over an iNGRV, but not over the iNGRV-DTP combination.

In summary, this survey suggested that an iNGRV-DTP combination vaccine appears to have high appeal due to the lack of extra cold chain or additional injection requirements, the large potential economic savings, and the possibility to avert an increased number of rotavirus-attributable deaths and cases.

## 6. Combination Vaccine Strategies

For an iNGRV to be combined with current pediatric childhood vaccines used in LMICs, the compatibility and stability of each of the antigens must be assessed. David Volkin and Sangeeta Joshi (Vaccine Analytics and Formulation Center, University of Kansas, USA) presented data on the compatibility of the three NRRV antigens in various pediatric vaccine combinations. Two NRRV-combination vaccines formulation options were explored: (1) hexavalent (P2-VP8 + pentavalent containing wcP, D, T, Hep B and Hib), and (2) bivalent (P2-VP8 + trivalent IPV). The P2-VP8 vaccine candidate is adsorbed to Alhydrogel, a commonly used vaccine aluminum salt adjuvant that improves immunogenicity. Because commonly used vaccine preservatives destabilize Alhydrogel-adjuvanted P2-VP8 antigens, including thimerosal and 2-PE, the P2-VP8 vaccine candidate in clinical trials is formulated with Alhydrogel and without preservatives.

The combination of P2-VP8 (formulated with Alhydrogel) with pentavalent vaccine (containing both Alhydrogel and Adjuphos aluminum adjuvants) results in partial desorption of P2-VP8, likely caused by wcP antigen and Adju-Phos. To work around this, the group combined P2-VP8 into a hexavalent formulation using only Alhydrogel and achieved >90% P2-VP8 adsorption. This approach, however, resulted in destabilization of P[6] component over time, indicating that P2-VP8 is destabilized by the antigens in the pentavalent formulation. Finally, a mixture of P2-VP8 and t-IPV resulted in P2-VP8 desorption from Alhydrogel with concomitant adsorption of the t-IPV to Alhydrogel. While this effect may be remedied by using a low phosphate buffer concentration, this results in t-IPV destabilization. In summary, further formulation development is required to achieve an optimized, stable hexavalent (P2-VP8-pentavalent) and bivalent (P2-VP8-IPV) combination vaccine.

Carl Kirkwood (BMGF) presented on potential vaccine combinations targeting viral diarrhea pathogens. Should combination of an iNGRV with existing childhood vaccines not prove to be technically or logistically feasible, there may be opportunities for new rotavirus-anchored combinations that target diarrheal disease. Although rotavirus is still the major cause of severe GE in children, there are several other viruses responsible for significant disease burden, including norovirus, adenovirus, sapovirus, and astrovirus, all of which warrant consideration for vaccine development.

The viability and success of this approach relies on multiple considerations, which include price-sensitivity of GAVI-transitioning countries, the potential that addressing non-bacterial causes of diarrhea may decrease antibiotic use and thereby help reduce AMR, the disease burden, and the prioritization of switching from an oral option to a parenteral option. The ideal vaccine platform (mRNA, subunit, etc.) for such a combination is not yet clear, but it is likely that there would be limited space for antigen display available in the product even using new platforms such as nanoparticles or mRNA, thereby limiting the number of viruses that may be targeted. In addition, there is a need to identify protective antigen(s) for many of these potential novel viral targets. Moreover, full efficacy studies will likely be required because there is no identified correlate of protection for either rotavirus or the other viruses of interest.

Bill Hausdorff (PATH) continued the conversation on challenges that confront a combination vaccine. Although there has been a great deal of work on understanding the value proposition of combination vaccines, there are three key challenges that combination vaccines face: (1) the absence of preferential policy recommendations to stimulate development, (2) the need to show geographic overlap of the diseases targeted by individual vaccine components, and (3) uncertainty about the need to demonstrate efficacy of each individual vaccine component. 

Regarding policy, the Strategic Group of Advisory Experts (SAGE)/WHO does not explicitly endorse the use of a combination vaccine over use of its individual vaccine components, and thus a manufacturer may be unsure of the return on making the considerable investment necessary to develop a combination. This is particularly relevant given recent analyses conducted by Linksbridge indicating that, based on the national stakeholder preferences described in an earlier presentation, an injectable rotavirus vaccine that is non-superior to the oral options, but combined with DTP, could theoretically take over the majority of the rotavirus market in Gavi support-eligible counties as well as non-Gavi MICs (assuming release by 2027) [52]. Assuming that such a combination is developed, policy considerations of global purchasing bodies are important and can have major implications on vaccine uptake. For example, every 5 years, Gavi assesses the landscape of vaccines that are already in advanced stages of development to determine its investment strategy. However, even Gavi’s endorsement of a combination does not guarantee that countries will prefer it to the separate components. Widespread adoption of the combination DTwcP-Hib-HepB did not occur until Gavi withdrew its financial support for the standalone HepB vaccine.

Combination vaccines may face imperfect geographic overlap of disease for each component. While geographic overlap is desirable, it may not be obligatory—for example, meningococcus A has not been seen in the US for decades but is included in the US’s meningococcal national immunization program. However, overlapping disease age-incidences is fundamental to the utility of a combination vaccine, because timing and numbers of doses of each component must be compatible.

Regarding efficacy, US FDA guidance on combination vaccines recommends evaluating the efficacy of individual vaccine components against their performance in combination. However, for vaccines where individual components do not have an existing standalone vaccine, there may be potential flexibility in the approval pathway. Such flexibility was apparent in the licensure requirements for multi-serotype or serogroup pneumococcal and meningococcal vaccines, where despite the absence of clear correlates of protection for most of the components, vaccine development and registration were able to proceed expeditiously. However, the approval pathway for a multi-pathogen combination vaccine today will likely face different requirements than the combination pneumococcal or meningococcal vaccines, despite them all being considered combinations.

## 7. WHO Roles and Support for Vaccine Development

Martin Friede (WHO, Geneva) spoke on the importance of looking beyond vaccine development to regulatory considerations for a future hypothetical next generation rotavirus vaccine. Even if a vaccine is approved by a country’s national regulatory authority (NRA), it must achieve WHO prequalification to be acquired and used by UNICEF and other vaccine-procurement agencies.

The WHO is currently piloting the Evidence Considerations for Vaccine Policy, driven by the Product Development for Vaccines Advisory Committee (PDVAC), and vaccine developers are advised to talk to PDVAC about the evidence required for inclusion in vaccine policy. In the case of rotavirus, there will need to be studies comparing any novel parenteral vaccine to the existing oral options, requiring significant time and financial resources. To aid this, it is important to create a global target product profile (TPP) for a parenteral rotavirus vaccine, so that there is global consensus. There may be challenges in communicating clear messages on the value of rotavirus vaccines, particularly when vaccines are introduced in populations with varying levels of disease burden, even if driven by environmental or geographic considerations (in this case, the lower effectiveness of oral vaccines in specific settings).

When considering cost and complexity of delivery of a parenteral vaccine, combination vaccines likely have an advantage over stand-alone vaccines as they reduce delivery costs and complexity. If a parenteral rotavirus vaccine is approved and policy formulated for its use, commercial production within regions of greatest need would be a huge step forward for decentralization of vaccine manufacturing. There are several key issues to this model, including workforce training, access to expertise, an existing national regulatory agency with WHO approval that can approve local products, sustainable access to the product, and a stable supply chain. Access to know-how may be addressed by technology transfer hubs. For mRNA vaccines and emerging technologies, this may be difficult as there are fewer global experts to recruit.

## 8. Broader Learnings and Conclusions

To close out the convening and recap key learnings, Gagandeep Kang (CMC Vellore, India), Harry Greenberg (Stanford University, USA), Miren Iturriza-Gómara (PATH), and Roger Glass (CDC, USA) participated in a panel discussion moderated by Duncan Steele (BMGF). The need for a more efficacious rotavirus vaccine, to achieve a high level of protection in LICs and LMICs, is clear. However, the group also agreed that there must be a continuous effort to expand coverage and reach unvaccinated children, where significant burden remains and for whom protection could be provided from the existing options as novel vaccines are being developed.

The use of a booster shot was reaffirmed to be an unlikely solution for increasing protection by rotavirus vaccination due to the relatively late ages targeted for the boost. A better solution is to find a more efficacious vaccine that can be combined with other childhood vaccines, even if parenterally administered. The lack of correlates of protection for rotavirus means efficacy studies for novel candidates must still rely on clinical outcomes and can therefore be prohibitively expensive to conduct. For this reason, it is important that groups continue to make efforts to find a reliable CoP for rotavirus protection. For vaccines that undergo clinical trials, whether or not they achieve WHO prequalification, there is likely to be information on mechanisms of action and CoPs that can be obtained from the clinical samples.

Given the current state of the field, future convenings and work on a next-generation rotavirus vaccine might explore a few key areas: (1) technical progress on next-generation rotavirus vaccine candidates, (2) understanding global regulatory considerations, both for a rotavirus standalone vaccine as well as any potential combination, (3) further detailed understanding of in-country considerations for rotavirus vaccine uptake, (4) progress on correlates of protection work, and (5) options for combining candidate rotavirus vaccines with vaccines that protect against other childhood diseases and/or causes of severe diarrhea.

## Figures and Tables

**Table 1:** Rotavirus vaccines licensed for global use, in clinical development, and selected vaccines in pre-clinical development.

Vaccine	Developer (Country)	Stage of Development
ROTARIX	GSK (Belgium)	WHO Prequalification
ROTAVAC	Bharat Biotech (India)	WHO Prequalification
ROTASIIL	Serum Institute of India (India)	WHO Prequalification
ROTATEQ	Merck (USA)	WHO Prequalification
LLR	Lanzhou Biologicals (China)	National approval (China)
Rotavin-M1 and Rotavin (liquid)	POLYVAC (Viet Nam)	National approval (Viet Nam)
UK-BRV hexavalent	Wuhan Institute (China)	Phase 3 (China)
LLR3 (human-lamb trivalent reassortant)	Lanzhou Biologicals (China)	Phase 3 (China)
RV3-BB	PT Bio Farma (Indonesia)	Phase 3 (Indonesia)
NRRV	PATH (USA)	Phase 3
MT-5625	Mitsubishi/Medicago (Japan/Canada)	Phase 1
IRV	Chinese Acad Med Sci/Henan CDC (China)	Phase 1
RV3-BB	MaxVax (China)	Preclinical (China)
BWV-301	Blue Water Vaccines/Cincinnati Children’s Hospital Medical Center (USA)	Preclinical
IRV CDC-9	US CDC (USA)	Preclinical
mRNA VP8	CureVac (Germany)	Preclinical
Rotavirus-norovirus particles	GIVAX Inc/Indiana University (USA)	Preclinical
VP4 *	Xiamen University/Innovax (China)	Preclinical

LLR = Lanzhou lamb rotavirus, BRV = bovine rotavirus vaccine (UK refers to the bovine G6P [5] strain), NRRV = non-replicating rotavirus vaccine, IRV = inactivated rotavirus vaccine, * = truncated VP4.

## Data Availability

Not applicable.
